# Neurodegeneration and the Circadian Clock

**DOI:** 10.3389/fnagi.2017.00170

**Published:** 2017-05-30

**Authors:** Suzanne Hood, Shimon Amir

**Affiliations:** ^1^Department of Psychology, Bishop’s UniversitySherbrooke, QC, Canada; ^2^Department of Psychology, Concordia UniversityMontreal, QC, Canada

**Keywords:** circadian rhythms, Alzheimer’s disease, Parkinson’s disease, Huntington’s disease, neurodegeneration, sleep, clock genes

## Abstract

Despite varied etiologies and symptoms, several neurodegenerative diseases—specifically, Alzheimer’s (AD), Parkinson’s (PD), and Huntington’s diseases (HDs)—share the common feature of abnormal circadian rhythms, such as those in behavior (e.g., disrupted sleep/wake cycles), physiological processes (e.g., diminished hormone release) and biochemical activities (e.g., antioxidant production). Circadian disturbances are among the earliest symptoms of these diseases, and the molecular mechanisms of the circadian system are suspected to play a pivotal, and possibly causal, role in their natural histories. Here, we review the common circadian abnormalities observed in ADs, PDs and HDs, and summarize the evidence that the molecular circadian clockwork directly influences the course of these disease states. On the basis of this research, we explore several circadian-oriented interventions proposed as treatments for these neurological disorders.

## Circadian Rhythms and Neurodegenerative Diseases

As life expectancy increases globally, the prevalence of neurodegenerative diseases mounts steadily. Worldwide, Alzheimer’s disease (AD), Parkinson’s disease (PD), and Huntington’s disease (HD) are among the most prevalent neurodegenerative diseases, and are associated with a significant burden for health care systems (Neurological Health Charities Canada, 2014, September). Despite the varied pathogenesis and diversity of symptoms among them, common to AD, PD and HD are disruptions of circadian rhythms, or the near 24-h cyclic fluctuations in a host of physiological and behavioral processes. A rapidly growing body of research suggests that disturbances in the circadian system precede the emergence of the characteristic cognitive and motor symptoms of these diseases by years (Kondratova and Kondratov, 2012; Hastings and Goedert, 2013; Videnovic et al., 2014a; Abbott and Videnovic, 2016; Mattis and Sehgal, 2016), and may contribute to their onset (Kondratova and Kondratov, 2012; Videnovic and Zee, 2015). Here, we provide a concise overview of the evidence linking the circadian system to these diseases, and examine circadian-oriented approaches to the treatment of AD, PD and HD.

### Circadian Rhythms and Cellular Clocks

The circadian system provides an adaptive mechanism for organisms to coordinate cellular processes, physiological functions and behaviors with the predictable 24-h cycle of light and dark on Earth (Bell-Pedersen et al., 2005). In humans, familiar examples of rhythms include daily patterns of sleeping and waking; the rise and fall of core body temperature; heart rate; blood pressure; and release of a wide variety of hormones, such as the nightly surge in melatonin from the pineal gland. The presence of an endogenous timing system in the body is clearly seen in conditions when predictable time-of-day cues are removed, yet near-24 h rhythms in these processes persist nonetheless (Arendt, 2012).

In mammals, the suprachiasmatic nucleus (SCN) houses the master circadian clock, and is found just dorsal to the optic chiasm. Inputs to the SCN from the retinohypothalamic tract provide information about daily light exposure to synchronize the endogenous clockwork to the external environment (Welsh et al., 2010). In turn, the SCN communicates time-of-day information by both synaptic and diffusible signals to a host of peripheral oscillators in a variety of brain regions and organs, such as heart, lungs, liver and adrenal glands. Thus, the SCN serves to coordinate the timing of a distributed network of clocks throughout the body (Mohawk et al., 2012). This coordination is vital for health and well-being: circadian desynchrony is already implicated in a number of disease states, including some cancers, metabolic diseases, and mood disorders such as bipolar disease and major depression (Roybal et al., 2007; McFadden et al., 2014; Stevens et al., 2014; Lucassen et al., 2016; Morris et al., 2016).

**Figure 1:**
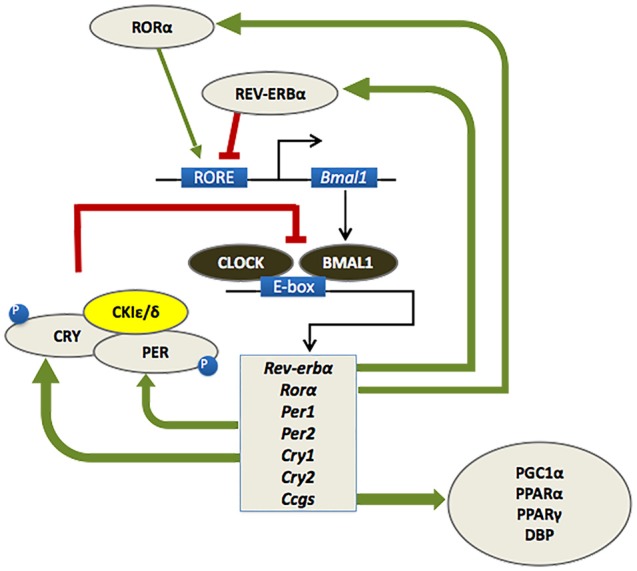
At a molecular level, the mammalian circadian clock is composed of a group of clock genes that regulate their own transcription and translation in a series of interlocking negative feedback loops. Heterodimers of the transcription factors BMAL1 and CLOCK drive the expression of the *Period* (*Per1/Per2*) and *Cryptochrome* (*Cry1/Cry2*) genes, the nuclear receptors retinoid-related orphan receptor (RORα) and REV-ERBα, and a number of downstream genes referred to as clock-controlled genes (CCGs). The protein products of the *Per* and *Cry* genes dimerize and inhibit the transcriptional activity of CLOCK-BMAL1. A number of kinases, such as casein kinase 1ɛ/δ (CK1 ɛ/δ), regulate the activity of PER-CRY dimers at a post-transcriptional level. RORα and REV-ERBα also regulate the transcription of BMAL1, whereby RORα promotes its expression, whereas REV-ERBα inhibits it. This cycle of clock gene expression completes in approximately 24 h (Huang et al., 2011; Mohawk et al., 2012).

As outlined in Figure 1, the circadian timekeeping mechanism is controlled at a cellular level by a group of genes that regulate their own transcription and translation over approximately 24 h via a series of interacting negative feedback loops (for a review see Mohawk et al., 2012). In addition to regulating their own levels of expression, “clock” genes serve as transcription factors for other genes which regulate a variety of functions, including cell division, metabolism, immune responses and oxidative processes (Duffield, 2003; Wilking et al., 2013). Importantly, mutations of the *bmal1* and *period* genes yield an accelerated aging phenotype in *Drosophila* and mice, with faster rates of tissue decline, impairments in cognitive function and shorter lifespan relative to age-matched wild type controls (Kondratov et al., 2006; Krishnan et al., 2009).

## Circadian Symptoms of Alzheimer’s, Parkinson’s and Huntington’s Diseases

Like other physiological processes, activity of the circadian system changes significantly across the lifespan (for recent reviews see Duffy et al., 2015; Hood and Amir, 2017), with a number of disruptions to rhythms such as the sleep/wake cycle and hormone release emerging in older adulthood. Importantly, some of these age-related disturbances in a number of rhythms resemble the circadian disturbances observed in AD, PD and HD, which are reviewed in the following sections. In noting these similarities, it is vital to recognize that differences exist in the severity and timing of the onset of circadian disturbances in sufferers of AD, PD and HD, compared to their occurrence during otherwise healthy aging. By distinguishing more clearly between changes in rhythms that reflect neurodegenerative processes and those that may not necessarily be pathological, we may be able to identify the development of disease more readily and, potentially, improve prospects for intervention and care.

### Sleep/Wake Rhythms

Disturbances in the sleep/wake rhythm are perhaps the most prominent circadian-related symptom in individuals affected by AD, PD, or HD. Nighttime sleep becomes increasingly fragmented as these diseases progress, while nocturnal activity levels and daytime sleepiness increase (Hatfield et al., 2004; Morton et al., 2005; Barone et al., 2009; Merlino et al., 2010; Weissová et al., 2016). In severe cases, minimal differences exist between day and night in bouts of activity and sleep (McCurry et al., 1999). These observations of poorly consolidated rest/activity patterns in humans are paralleled by animal models of each disease state (Morton et al., 2005; Vezoli et al., 2011; Loh et al., 2013; Fifel and Cooper, 2014; Long et al., 2014; Graybeal et al., 2015). In addition, behavioral sleep disorders, such as restless leg syndrome and rapid eye movement behavior disorder (RBD) are highly comorbid with PD (Comella, 2007; Barone et al., 2009). Together, these declines in the normal sleep/wake rhythm and in the quality of sleep are identified as among the most disruptive symptoms of these diseases, and have a profoundly negative impact on quality of life (Barone et al., 2009). Furthermore, they are cited as primary reasons for entering individuals with a neurodegenerative illness into residential care facilities (Pollak and Perlick, 1991; Bianchetti et al., 1995).

### Melatonin and Cortisol Rhythms

Disturbances have been documented in the circadian rhythms of melatonin and cortisol release in AD, PD and HD. Common to each is a flattening of the melatonin rhythm, such that the normal nighttime peak is suppressed relative to healthy, age-matched controls (Mishima et al., 1999; Wu et al., 2003; Breen et al., 2014; Kalliolia et al., 2014; Videnovic et al., 2014b; although see Aziz et al., 2009). A decline in the peak of nighttime melatonin release has also been observed in individuals exhibiting pre-clinical cognitive symptoms of dementia (Wu et al., 2003; Waller et al., 2016), and this decline appears to correlate positively with level of daytime sleepiness (Videnovic et al., 2014b).

Changes in the rhythm of cortisol release have also been observed, although these changes are somewhat more varied compared with those in melatonin. The normal cortisol rhythm rises in the early morning, with the peak occurring near waking and the nadir in the late evening (Touitou and Haus, 2000). Minimal change in this rhythm has been observed in individuals with suspected AD or dementia (Hatfield et al., 2004; Waller et al., 2016; although see Giubilei et al., 2001; and Hartmann et al., 1997). In both PD and HD, the diurnal pattern of cortisol release remains rhythmic, yet the total daily amount of cortisol released is elevated (Hartmann et al., 1997; Aziz et al., 2009).

### Core Body Temperature Rhythm

The human core body temperature rhythm rises throughout the day to peak in the early evening, then falls throughout the night to reach its nadir in the early morning (Van Someren, 2000). Studies of individuals with AD indicate a delay in the peak of this rhythm and a decrease in its amplitude (Satlin et al., 1995; Harper et al., 2005). In PD, only the amplitude of the rhythm appears to be decreased. This change is attributed to a lowering of peak body temperature relative to healthy age-matched controls (Pierangeli et al., 2001; Zhong et al., 2013). A profound reduction in the amplitude of the temperature rhythm has also been documented in rodent models of HD (Kudo et al., 2011; Fisher et al., 2013).

### Mood and Behavior Rhythm

A rhythm in mood and emotional volatility reportedly emerges as neurodegenerative conditions progress. This “sundown syndrome” comprises a daily pattern of increased agitation, emotional volatility, and aggression that peaks in the late afternoon or evening (for review see Bachman and Rabins, 2006). This syndrome is not formally recognized as a clinical condition—indeed, dispute exists as to what behavioral features it includes, and whether increased behavioral disturbances during this time of day truly reflect a clinical phenomenon or a confounding influence, such as reporting bias from caregivers (e.g., Bliwise et al., 1993; Yesavage et al., 2003). However, a number of reports suggest that a small but notable proportion of elderly individuals with dementia do exhibit a predictable diurnal pattern of behavioral and emotional disturbance, particularly among those with severe symptoms (Gallagher-Thompson et al., 1992; Martin et al., 2000). The factors contributing to the expression of agitated behaviors are unknown, but some evidence suggests that this pattern is not a direct consequence of sleep loss (Volicer et al., 2001).

## Neurodegenerative Diseases and Clock Gene Expression

Evidence from individuals with AD, PD, or HD and animal models of each disease state indicate abnormalities in the rhythms of *bmal1* and *per2* expression. In AD, the pattern of change observed in *bmal1* mRNA expression is complex. In several brain regions and peripheral tissues, *bmal1* mRNA expression remains rhythmic; however, the temporal phase relationships among these tissues differ compared with healthy controls (Cermakian et al., 2011; Weissová et al., 2016). In the pineal gland, the rhythms of *bmal1, per1* and *cry1* mRNA are lost (Wu et al., 2006). In PD, the *bmal1* transcription rhythm in blood cells is blunted in amplitude (Cai et al., 2010; Breen et al., 2014). Furthermore, rodent models of PD exhibit a blunting of rhythmic *per2* mRNA and PER2 protein expression in several brain regions downstream of SCN control and in peripheral tissues. For example, loss of dopaminergic innervation to the striatum abolishes the rhythmic expression of PER2 protein in this region (Hood et al., 2010; Gravotta et al., 2011). Similarly, the normal rhythms of *per2* mRNA expression in both central and peripheral tissues are disrupted in rodent models of HD (Morton et al., 2005; Maywood et al., 2010).

## Does A Faulty Circadian Clock Cause Neurodegenerative Disease?

Given the prevalence of rhythm abnormalities in neurodegenerative diseases, circadian disturbances are increasingly regarded as harbingers of neurodegeneration (e.g., Videnovic and Zee, 2015; Mattis and Sehgal, 2016). Consistent with this idea, several prospective studies have identified excessive daytime sleepiness (Abbott et al., 2005; Bonanni et al., 2005), daytime activity fragmentation (Tranah et al., 2011), and sleep behavior disorders (Iranzo et al., 2013; Schenck et al., 2013; Postuma et al., 2015) as independent predictors of AD, PD and cognitive impairments associated with dementia. In the case of RBD, the vast majority of affected individuals appear to be at risk of developing PD or a related synucleinopathy, particularly if additional non-motor risk factors are also exhibited (Iranzo et al., 2013; Schenck et al., 2013; Postuma et al., 2015).

Are these circadian disruptions a consequence of neurodegeneration affecting clockwork mechanisms in the brain and periphery, or do malfunctioning endogenous clocks directly contribute to disease progression? It is clear that prolonged disruption of normal circadian rhythms yields a variety of negative effects on health via mechanisms including widespread impact on gene transcription and pro-inflammatory processes (Archer and Oster, 2015; Lucassen et al., 2016), which may exacerbate the progress of these pathologies. However, a number of findings indicate that the circadian system may in fact play a more direct role in the etiology of neurodegenerative diseases. For example, single nucleotide polymorphisms of *bmal1* and *per1* are associated with increased risk of PD (Gu et al., 2015). Furthermore, clock genes regulate the expression of other genes directly implicated in neurocognitive disorders such as AD (Panda et al., 2002; Duffield, 2003; Li et al., 2013). For example, the *presenilin-2* gene, which regulates levels of beta amyloid peptide and is linked to familial early onset AD (Levy-Lahad et al., 1995; Giri et al., 2016), is rhythmically expressed in SCN (Esler and Wolfe, 2001; Panda et al., 2002). In peripheral tissues, CLOCK:BMAL dimers regulate the expression of *presenilin-2* via transcriptional and post-transcriptional mechanisms (Bélanger et al., 2006). These findings suggest a causal link between clock genes and molecular factors that confer risk of neurodegeneration. To our knowledge, however, no experimental studies have yet demonstrated that manipulation of clock genes affects the expression of *presenilin-2* in brain.

Degenerative changes within the SCN itself may play a contributory role in these disease states, although evidence supporting this possibility is not entirely consistent. Some post-mortem studies of brain tissue from sufferers of AD indicate loss of hypothalamic tissue that includes cells in the SCN, a reduction in the expression of the neuropeptides AVP and VIP (Swaab et al., 1985; Stopa et al., 1999), and a decrease in the expression of the melatonin receptor MT1 (Wu et al., 2007; however, see Wang et al., 2015). Rodent models of HD exhibit reduced spontaneous cell firing in the SCN compared with controls (Kudo et al., 2011; although see Pallier et al., 2007), yet no change in SCN cell number (Fahrenkrug et al., 2007). Although coordinated SCN cell firing appears to diminish as a normal part of aging (Farajnia et al., 2012), this reduction occurs at a prematurely young age (3 months) in HD rodent models (Kudo et al., 2011). Given that other hypothalamic structures degenerate in AD, PD and HD (Shan et al., 2015), it is possible that structural changes to the master clock may be a consequence of the progressive course of tissue destruction in each disease state, rather than precede disease onset. Nevertheless, any dysfunction of the master clock is likely to worsen the symptoms of these diseases through downstream effects on peripheral oscillators. Consistent with this idea, levels of beta amyloid peptide in human cerebrospinal fluid have been found to correlate positively with sleep fragmentation (Ju et al., 2013).

Compelling evidence suggests that the circadian system may contribute to neurodegenerative disease states through its involvement in regulating cellular responses to oxidative stress (Kondratova and Kondratov, 2012). Oxidative stress is suspected as a causal factor of neuronal damage, cell death, and mitochondrial dysfunction observed in AD, PD and HD (for a review see Grimm et al., 2011). Clock genes such as *bmal1* have been directly implicated in cellular antioxidant responses through downstream regulation of antioxidant response element transcription factors (Lee et al., 2013). Rodents with selective knockouts of *bmal1* or the *period* genes (*per1* and *per2*) exhibit significantly higher rates of oxidative damage in tissues compared with age-matched wild type controls (Kondratov et al., 2006; Jang et al., 2011; Lee et al., 2013). The circadian clock may also regulate oxidative stress via rhythmic release of melatonin, which is an effective free radical scavenger (Reiter et al., 2002). These findings imply that abnormal operation of the molecular clock may create cellular conditions whereby harmful by-products of metabolism and DNA replication accumulate, and mitochondrial damage may develop. In turn, these conditions may promote the pathogenesis of neurodegenerative disease states.

## Circadian-Oriented Interventions in Neurodegenerative Disease

If the circadian system is indeed a contributor to neurodegenerative disease, it follows that therapeutic interventions targeting the circadian clock could mitigate symptoms, or perhaps even retard the course of the disease itself. To this end, a number of circadian-oriented therapies have been investigated for AD, PD and HD.

One of the most frequently explored examples of this kind of intervention is the use of bright light therapy. Previous evidence has shown that institutionalized older adults may have very little daily exposure to bright light, particularly those with severe symptoms of dementia (Ancoli-Israel et al., 1997; Shochat et al., 2000). Given the profound effect of light exposure in regulating the timing of the master clock, a number of studies have evaluated whether timed bright light exposure has any beneficial effect on the course of neurodegeneration or its symptoms. To date, results have been mixed (Forbes et al., 2014). Overall, timed light exposure appears to modestly improve the regulation of the circadian system in individuals with neurodegenerative disease. In the case of AD, some positive but short-lived benefits have been reported for timed daily exposure to bright light on the consolidation of activity rhythms in elderly adults with AD (Ancoli-Israel et al., 2003; McCurry et al., 2011) and severe dementia (Ancoli-Israel et al., 2002). In PD, daily light exposure improves sleep/wake rhythms through reducing daytime sleepiness and increasing daytime activity (Videnovic et al., 2017). However, it remains unclear whether timed light exposure lessens cognitive or motor skill decline over time. Although light exposure regimens have yielded some short-term improvements in activities of daily living in individuals with AD or PD, there is not yet sufficient evidence to conclude any long-lasting cognitive or motor benefits of this intervention (Paus et al., 2007; Forbes et al., 2014).

Timed administration of melatonin has been investigated for its therapeutic potential in AD, PD and HD. As shown *in vitro* and in animal models, melatonin has antioxidant and apoptotic properties (Reiter et al., 2002; Wang et al., 2011), and appears to prevent the formation of alpha-synuclein protein aggregations (the primary protein component of Lewy bodies; Ono et al., 2012). However, in randomized controlled clinical trials in humans, the effects of melatonin supplements on sleep quality and activity rhythms have been inconsistent. In individuals with PD, daily doses of melatonin did not improve sleep quality, but were associated with improved self-report measures of sleeping (Medeiros et al., 2007). In trials involving individuals with suspected AD, modest improvements in sleep quality (reduced sleep latency, improved sleep efficiency) and increased total sleep time were observed in some cases (Asayama et al., 2003; Riemersma-van der Lek et al., 2008), particularly when melatonin treatments were combined with daily bright light therapy (Riemersma-van der Lek et al., 2008). However, other trials failed to identify any effects on circadian rhythms of activity, sleep, or cognitive symptoms (Singer et al., 2003; Gehrman et al., 2009; reviewed in Urrestarazu and Iriarte, 2016). A beneficial effect of melatonin supplements has been reported for behaviors associated with sundown syndrome, but this effect has not been found consistently in randomized controlled trials (Riemersma-van der Lek et al., 2008; de Jonghe et al., 2010).

The lack of evidence that timed light exposure and melatonin administration improve the non-circadian symptoms of AD, PD and HD would seem to undermine the idea that the circadian system contributes to the etiology of these neurodegenerative diseases. It is likely, however, that some methodological inconsistencies across trials have contributed to these inconclusive findings. For example, studies evaluating light exposure have varied markedly in the intensity of light used; the timing of light exposure; and clinical characteristics of the participants (Forbes et al., 2014). Similarly, variability in the dosage, timing of administration, and characteristics of the sample under study may have contributed to the inconsistency of findings regarding the impact of melatonin (Urrestarazu and Iriarte, 2016). Furthermore, to our knowledge, no longitudinal studies have yet evaluated whether circadian-oriented interventions moderate the long-term progression of neurodegenerative disease symptoms. Careful consideration of these methodological details and the incorporation of long-term follow-up intervals would be of benefit in the design of future research. As the circadian features of neurodegenerative diseases may reflect a desynchronization of tissue oscillators downstream of SCN control from the master clock, an additional avenue of therapeutic intervention concerns the powerful influence of timed food delivery in the entrainment of circadian rhythms (Maywood et al., 2010; Cermakian et al., 2011). The circadian system retains sensitivity to food as a time cue, or *zeitgeber*, over the course of healthy aging (Walcott and Tate, 1996). Given that timed food delivery is a highly potent *zeitgeber* and does not exert its entrainment effects via the SCN (Boulos et al., 1980), it is possible that timed feeding or metabolic cues could serve to re-synchronize disrupted circadian timing (for a review see Kent, 2014). Indeed, some evidence suggests that food intake patterns in individuals with suspected AD or associated dementias vary with the progression of illness. For example, institutionalized elderly with symptoms of AD tend to consume less food in the afternoon and evening compared with those without symptoms of AD, and breakfast becomes the primary meal providing the greatest amount of energy intake for the day (Young and Greenwood, 2001).

Recent studies suggest that imposing restricted meal times could mitigate some of the circadian symptoms of neurodegeneration. In the R6/2 rodent model of HD, the restriction of food access to a 6-h window in the light phase restored a rhythm of locomotor activity and altered clock gene expression patterns in liver, compared with wild type controls (Maywood et al., 2010). The use of a dark-phase restricted feeding schedule also appears to delay the developmental onset of the HD phenotype in R6/2 mice, and increases core body temperature compared with wild type controls (Skillings et al., 2014). Further research into the effects of timed food restriction using animal models of AD and PD would be valuable to pursue.

## Conclusions

Taken together, a growing body of evidence strongly implicates the circadian system in the onset and expression of AD, PD and HD. Disruptions to normal rhythmic processes are increasingly recognized as characteristic features of these disease states, and these disruptions may serve as early indicators of developing pathology. At the molecular level, clock genes regulate a number of genes and biochemical processes that contribute directly to neurodegeneration. Although it is currently unclear whether the circadian system plays a causal role in pathogenesis, further research may clarify this relationship. The advancement of knowledge on this subject may foster the development of screening tools to identify individuals at early stages of neurodegeneration, and may perhaps open a new realm of therapeutic interventions. Given the projected increase in the prevalence of neurodegenerative diseases in the coming years (Sosa-Ortiz et al., 2012), these advancements would be both timely and welcome.

## Author Contributions

SH and SA wrote the article.

## Conflict of Interest Statement

The authors declare that the research was conducted in the absence of any commercial or financial relationships that could be construed as a potential conflict of interest.
